# Musculoskeletal Pain in Parkinson's Disease

**DOI:** 10.3389/fneur.2021.756538

**Published:** 2022-01-21

**Authors:** Jun Li, Ben-Fan Zhu, Zhu-Qin Gu, Hui Zhang, Shan-Shan Mei, Shao-Zhen Ji, Shu-Ying Liu, Chao Han, Huai-Zhen Chen, Piu Chan

**Affiliations:** ^1^Department of Neurology, The First Affiliated Hospital of Anhui University of Traditional Chinese Medicine, Hefei, China; ^2^Department of Neurology, Neurobiology, and Geriatrics, Beijing Institute of Geriatrics, Xuanwu Hospital of Capital Medical University, Beijing, China; ^3^Department of Pain, The First Affiliated Hospital of Anhui Medical University, Hefei, China; ^4^Key Laboratory for Neurodegenerative Diseases of the Ministry of Education, Beijing Key Laboratory for Parkinson's Disease, Parkinson's Disease Center of Beijing Institute for Brain Disorders, Beijing, China; ^5^National Clinical Research Center for Geriatric Disorders, Beijing, China; ^6^Clinical and Research Center for Parkinson's Disease, Capital Medical University, Beijing, China; ^7^Advanced Innovation Center for Human Brain Protection, Capital Medical University, Beijing, China

**Keywords:** Parkinson's disease, musculoskeletal pain, pain, dopamine deficiency, risk factor

## Abstract

**Background:**

Musculoskeletal pain is commonly experienced in patients with Parkinson's disease (PD). Few studies have investigated the clinical characteristics and risk factors associated with musculoskeletal pain.

**Objectives:**

To investigate the distribution, clinical characteristics, and factors associated with musculoskeletal pain in a large sample of patients with PD.

**Methods:**

We enrolled 452 patients from two clinics and used a standardized questionnaire to collect demographic and clinical information. Musculoskeletal pain was diagnosed based on the Ford Classification System, and pain severity was assessed with the numeric rating scale (NRS). Multivariate regression models explored the association between clinical features of PD and quality of life and pain.

**Results:**

Two hundred and six patients (45.58%) reported musculoskeletal pain, typically in their lower limbs and backs. Levodopa resulted in a ≥30% reduction in pain intensity scores in 170 subjects. Female sex (odds ratio [OR], 1.57; 95% CI, 1.07–2.29) and Levodopa-equivalent daily doses (LEDDs; OR, 3.35; 95% CI, 1.63–6.59) were associated with an increased risk for musculoskeletal pain. Pain duration (*p* = 0.017), motor symptoms (*p* < 0.001), and depression (*p* < 0.001) were significantly associated with quality of life.

**Conclusions:**

The lower limbs and back are common sites of musculoskeletal pain in patients with PD, and up to 82.52% of patients were responsive to Levodopa. Female sex and LEDDs are associated with musculoskeletal pain, suggesting that dopamine deficiencies, and not the motor and non-motor impairment, might be the most critical baseline risk factor of musculoskeletal pain.

## Introduction

Pain is an important non-motor symptom of Parkinson's disease (PD), with an estimated prevalence of 40–83% (1). Pain can occur at any stage of PD, even before clinical diagnosis, and adversely affects the quality of life (QoL) of the patients (2, 3). There is no consensus among researchers on the classification of pain in PD. The most commonly used classification proposed by Ford et al. includes five subtypes: musculoskeletal pain (MSP), radicular/neuropathic pain, dystonia pain, akathisia pain, and central pain (4). MSP, as the most common subtype of pain in PD, has consequently attracted increasing attention. It is defined as pain in the muscles or joints and which may be related to poor posture. MSP can present as aches, cramping, or muscle stiffness and is often relieved by movement or improved with Levodopa (4).

Musculoskeletal pain is the cause of pain in 40–90% of patients with PD (2, 5). However, few studies have investigated the clinical characteristics and other factors associated with MSP. Previous studies have shown that MSP was attributable to motor symptoms in patients with PD, such as parkinsonian rigidity, increased stiffness, and reduced mobility (2, 4). In contrast, another study has reported that the severity of MSP does not correlate with the severity of motor symptoms and only weakly correlates with the severity of motor complications, autonomic symptoms, anxiety, and depression (6).

It is well-known that Levodopa is the most effective treatment for the motor symptoms of PD and can also alleviate MSP (2). However, the relationship between MSP, motor symptoms, and Levodopa therapy remains unclear. Pain in the lower back and pain due to frozen shoulder are highly prevalent among patients with PD (7); however, few studies have examined the distribution of pain sites and the response rate of MSP to Levodopa. Further, although pain is known to adversely affect QoL (6, 8), it remains unclear whether MSP, as a specific type of pain in PD, results in a decreased QoL.

This study investigates the clinical characteristics and associated factors of MSP to better understand the etiology of MSP in a large sample of patients with PD in China.

## Methods

### Ethics Approval

All the study procedures were performed following the principles of the Declaration of Helsinki and the institutional review boards of the Xuanwu Hospital (Beijing, China; support no.: [2019]024) and the First Affiliated Hospital of the Anhui University of Traditional Chinese Medicine (Hefei, China; approval no.: 2019AH-13). Each participant provided written informed consent.

### Patients

Consecutive outpatients with idiopathic PD who visited the movement disorders clinic of Xuanwu Hospital of Capital Medical University and the Neurology Department of the First Affiliated Hospital of the Anhui University of Traditional Chinese Medicine between July 2017 and January 2020 were enrolled. The inclusion criteria were (a) idiopathic PD diagnosed according to the 2015 Movement Disorder Society (MDS) clinical diagnostic criteria (9); (b) age 18–85 years old; and (c) Hoehn and Yahr stages of I–V (10). Patients with incomplete assessments and those with cognitive dysfunction as defined by a Mini-Mental State Examination score (MMSE) ≤24 were excluded. PD was diagnosed by senior neurologists who were experts in movement disorders.

Musculoskeletal pain was diagnosed by the Ford Classification System (4) for pain according to the diagnostic codes of the International Classification of Diseases, Tenth Revision, Clinical Modification: R52.901 (generalized body pain), R51.x00 (headache), M79.600x011 (shoulder pain), M54.200 (neck pain), M54.503 (back pain), M79.600x021 (upper arm pain), M79.600x051 (lower limb pain), M79.601 (hand pain), M25.507 (ankle pain), M79.604 (foot pain), M25.501 (shoulder joint pain), M25.502 (elbow joint pain), M25.503 (wrist joint pain), M25.504 (hand joint pain), M25.505 (hip joint pain), M25.506 (knee joint pain), M25.507 (ankle joint pain), M79.602 (foot joint pain), M79.101 (shoulder muscles pain), M79.102 (upper arm muscles pain), M79.103 (forearm muscles pain), M79.104 (hand muscles pain), M79.105 (pelvic muscles pain), M79.106 (thigh muscles pain), M79.107 (calf muscles pain), M79.108 (ankle muscles pain), and M79.109 (foot muscles pain). The inclusion criteria were (a) idiopathic PD with MSPand (b) pain relieved by mobility or improved with Levodopa (4). Exclusion criteria included (a) trauma to the affected joints and muscles in the preceding 3 months; (b) pain due to peripheral neuropathy detected by electrophysiological examinations; (c) comorbid conditions other than PD, which can cause pain, such as cancers, obvious lumbar disc herniation, severe lumbar spinal canal stenosis, rheumatoid arthritis, apparent osteoarthritis, or osteoporotic fracture, based on laboratory or imaging findings and aggravated by activity. A senior pain specialist reconfirmed MSP.

### Pain Sites and Assessments

All patients were asked to indicate the distribution of pain sites, duration of pain, and rate average pain intensity over the past 3 months on an 11-point numeric rating scale (NRS) (11, 12), with 0 indicating the absence of pain and ten indicating the most intense pain imaginable. The patients reported NRS scores in both the ON and OFF states of motor function on the same day. NRS scores were then transformed into WHO severity stages of “mild” (pain scores of 1–3), “moderate” (pain scores of 4–5), and “severe” (pain scores of 6–10), respectively (13). MSP responsiveness to Levodopa was defined as a ≥30% reduction in NRS scores from baseline, 2 h after taking the medication (12). Data regarding multiple types of pain were also recorded. The ID-pain scale detected central parkinsonian pain.

### Other Clinical Assessments

We used the MDS-Unified PD Rating Scale (MDS-UPDRS) to measure motor symptoms in the ON and OFF states. The Hamilton Depression Scale (HAMD), Hamilton Anxiety Scale (HAMA), PD Sleep Scale (PDSS), and PD Questionnaire (PDQ-39) were used to assess the severity of depression, anxiety, sleep quality, and QoL, respectively. The type and dose of PD medications at the time of assessment were recorded. Levodopa-equivalent daily doses (LEDDs) were calculated using the formulae described by Tomlinson et al. (14). The uses of analgesic medications were also recorded.

### Statistical Analyses

Statistical analyses were performed with SPSS version 23 (IBM, Armonk, NY, USA). Statistical significance was defined as *p* < 0.05. Clinical and demographic data were represented as mean ± SD, median with 25–75% interquartile range (IQR), or number (%). The Shapiro–Wilk test showed that our data for NRS scores (ON/OFF state) are not normally distributed. We used the non-parametric Wilcoxon signed-rank test to compare the NRS scores reported in the ON state with those reported in the OFF state. We calculated the proportion of pain in the ON/OFF state for every pain severity category in the MSP group.

Each patient was assigned 1 point if MSP was present or 0 if absent. The cut-off scores of HAMA, HAMD, and PDSS are 7 points for anxiety, 8 for depression, and 105 for sleep disorders, respectively. We used a *t*-test for continuous variables and the χ^2^-test for categorical variables to compare the patients who reported MSP and those who did not. The Box-Tidwell method was used to evaluate the linear relationships between continuous independent variables and the logit transformed dependent variable. The variables that exhibited significant between-group differences in these univariate analyses were entered into a multivariable binary logistic regression model with stepwise (forward) to calculate odds ratios (ORs) and 95% CIs to identify predictors of MSP.

To investigate the PDQ-39 domains associated with MSP, we first conducted a Pearson correlation analysis to determine candidate factors among the continuous variables. Then, we used multiple linear regression analysis to identify the genuinely relevant variables, and the regression analyses were corrected for the potential confounders.

## Results

### Patient Characteristics

A total of 452 patients were included in the analyses. The demographics and clinical characteristics of the study population are described in Table 1. A total of 206 patients (45.58%) reported MSP. Compared to the non-MSP group, the MSP group was older (63.04 ± 9.85 vs. 61.27 ± 10.74; *p* = 0.046), had a higher proportion of females (52.19 vs. 42.28%; *p* = 0.029), a longer disease duration (4.52 ± 3.26 vs. 3.83 ± 2.81; *p* = 0.01), higher MDS-UPDRS part I scores (8.96 ± 5.12 vs. 7.15 ± 4.80; *p* < 0.001), and higher doses of LEDDs (0.79 ± 0.31 vs. 0.71 ± 0.24; *p* = 0.001). There were no significant differences in the MDS-UPDRS part III, MDS-UPDRS part IV, HAMD, HAMA, PDSS, PDQ scores, and the Hoehn and Yahr scale between groups.

**Table 1:** Demographics and clinical characteristics of the study population.

**Variables**	**Patients with MSP (*n =* 206)**	**Patients without MSP (*n =* 246)**	***P-*value**
Age, years	63.04 ± 9.85	61.27 ± 10.74	0.046
Sex (female, %)	52.91%	42.28%	0.029
PD duration, years (mean)	4.52 ± 3.26	3.83 ± 2.81	0.01
MDS-UPDRS part I	8.96 ± 5.12	7.15 ± 4.80	<0.001
MDS-UPDRS part III (ON state)	19.12 ± 9.30	19.24 ± 10.83	0.842
MDS-UPDRS part III (OFF state)	33.99 ± 16.96	32.00 ± 17.54	0.287
MDS-UPDRS part IV	1.80 ± 3.52	1.28 ± 2.72	0.075
Hoehn and Yahr stage (ON state)	2.18 ± 0.78	2.23 ± 0.83	0.379
Hoehn and Yahr stage (OFF state)	2.40 ± 0.73	2.40 ± 0.81	0.782
HAMD			
HAMD <8	3.58 ± 2.11	10.62 ± 7.35	0.192
HAMD≥8	10.62 ± 7.35	10.62 ± 7.35	0.331
HAMA			
HAMA <7	10.62 ± 7.35	10.62 ± 7.35	0.376
HAMA≥7	10.62 ± 7.35	10.62 ± 7.35	0.870
PDSS			
PDSS <105	10.62 ± 7.35	10.62 ± 7.35	0.785
PDSS ≥105	10.62 ± 7.35	10.62 ± 7.35	0.778
PDQ-39	31.47 ± 18.46	31.87 ± 22.52	0.990
LEDD, g	0.79 ± 0.31	0.71 ± 0.24	0.001
NRS score (ON) (range)	0(0,2)	NA	NA
NRS score (OFF) (range)	5.5 (3,7)	NA	NA
Pain duration, years (mean)	3.25 ± 2.45	NA	NA

*All the continuous variables are expressed as number (percentage), mean ± standard deviation or median (interquartile range, 25–75%)*.

*HAMA, Hamilton Anxiety Scale; HAMD, Hamilton Depression Scale; MDS-UPDRS, Movement Disorder Society Unified PD Rating Scale; MSP, musculoskeletal pain; NRS, numeric rating scale; PD, Parkinson's disease; PDQ-39, PD Questionnaire; PDSS, PD Sleep Scale; LEDD, Levodopa-equivalent daily dose*.

**Figure 1:**
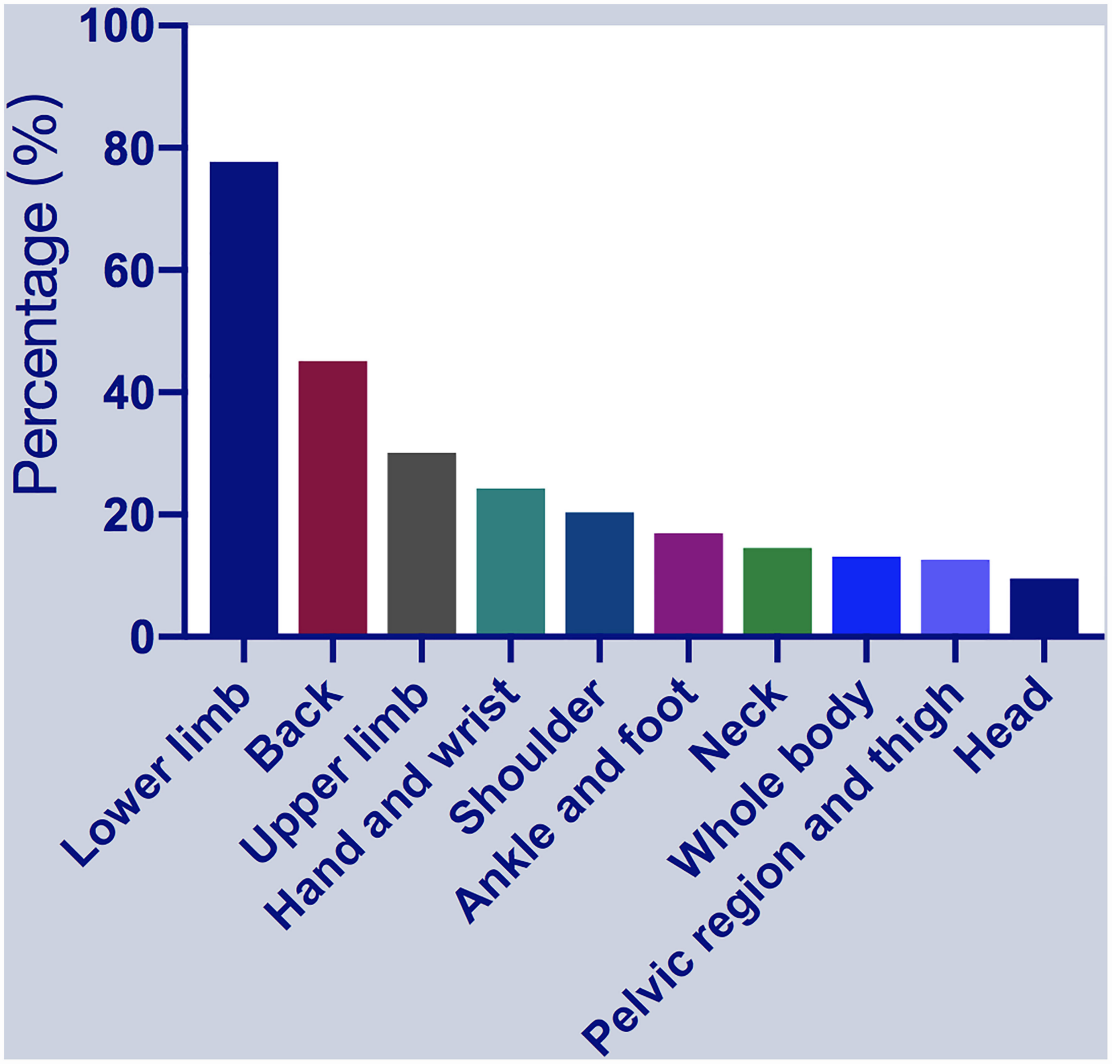
Percentages of patients with musculoskeletal pain (MSP) who reported pain at specific locations. MSP, musculoskeletal pain.

The reported sites of MSP included the lower limbs (*n* = 160; 77.67%), upper and lower back (*n* = 93; 45.15%), upper limbs (*n* = 62; 30.09%), hands and wrists (*n* = 50; 24.27%), shoulders (*n* = 42; 20.39%), ankles and feet (*n* = 35; 16.99%), neck (*n* = 30; 14.56%), whole-body (*n* = 27; 13.11%), pelvic region and thighs (*n* = 26; 12.62%), and head (*n* = 25; 9.54%) (Figure 1). Patients reported soreness, cramps, or dull aches, with associated itching, burning, tingling, or crawling sensations. However, some subjects had difficulty to accurately describe the types of pain, particularly in the OFF state. Of the 206 patients with MSP, 62 (30.09%), 87 (42.23%), and 57 (27.67%) reported mild, moderate, and severe pain severity stages in the OFF state, respectively; 22 (10.68%), 21 (10.19%), and 5 (2.43%) reported mild, moderate, and severe pain severity stages in the ON state, respectively. Most patients (164, 79.61%) experienced two or multiple pain sites, while 42 patients (20.39%) reported only one site of MSP. Regarding other pain types, akathisia, dystonia, radicular/neuropathic, and central pain were reported by 47, 42, 23, and 23 patients. Thirty-five (16.99%) patients had previously taken painkillers due to unbearable pain, but none were taking painkillers on the assessment day.

All patients received antiparkinsonian drugs. Of the 206 patients with MSP, 170 (82.52%) reported that MSP was responsive to Levodopa, as indicated by reducing pain with at least 30% improvement over a short period, whereas 36 patients (17.48%) showed no response. Of the non-responsive patients, one reported that MSP was worsened by Levodopa and alleviated when the Levodopa dose was reduced. In general, the NRS scores recorded in the ON state after Levodopa treatment (median, 0; IQR, 0–2) were lower than those recorded in the OFF state before Levodopa treatment (median, 5.5; IQR, 3–7; *p* < 0.001).

### Variables Associated With MSP

**Figure 2:**
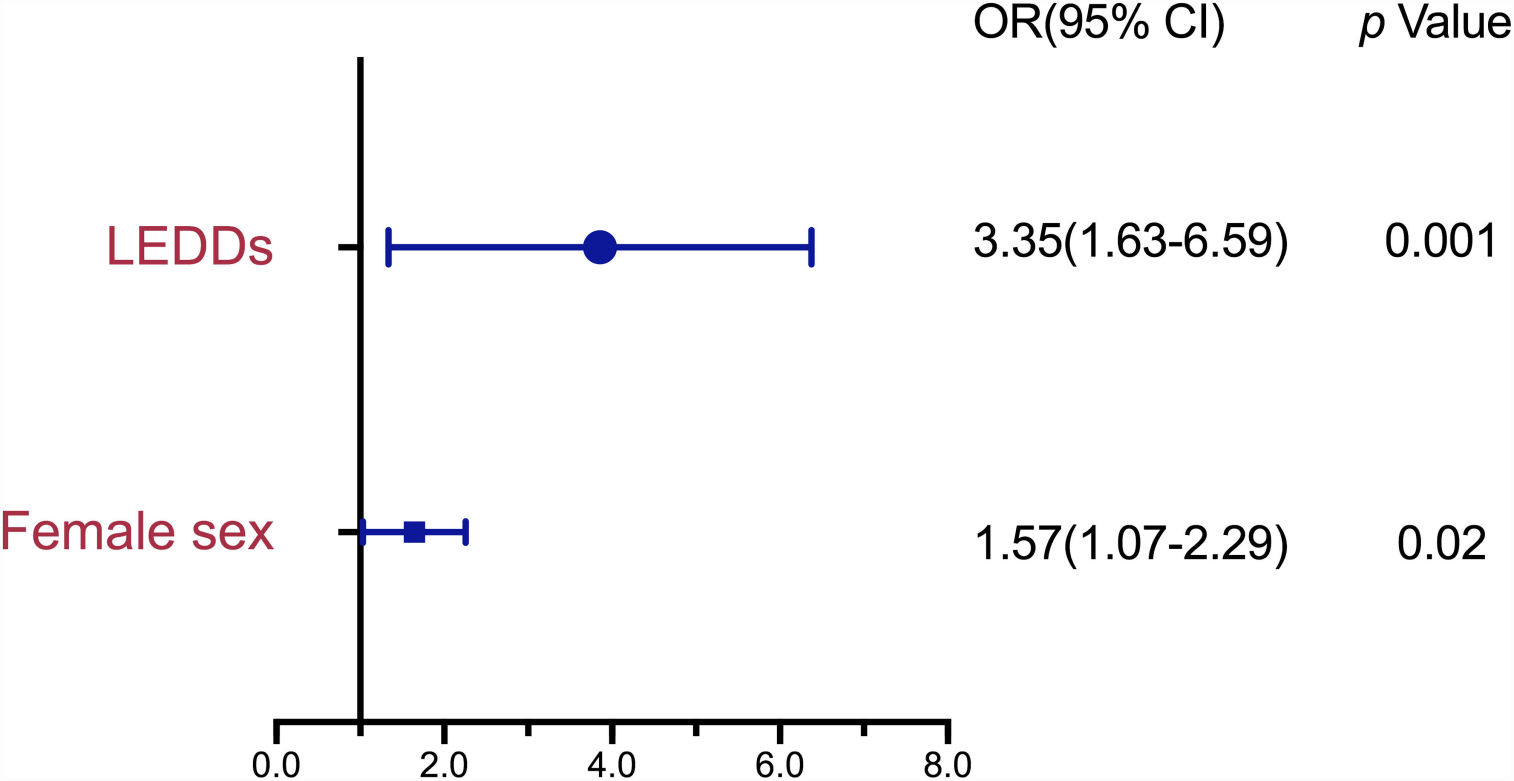
Risk Factors associated with MSP in patients with Parkinson's disease (PD) by binary logistic regression analysis. MSP, musculoskeletal pain; PD, Parkinson's disease; OR, odds ratio; CI, confidence interval; LEDD, Levodopa-equivalent daily dose.

Figure 2 shows the results of the logistic regression analysis of variables identified as potential risk factors of MSP. The candidate variables entered into the logistic regression model were female sex, PD durations, MDS-UPRDS I, and LEDDs. As the MDS-UPRDS I includes a pain-related item (item 1.9), we exclude this item from MDS-UPDRS I score and analyze it without this item. The variables that achieved statistical significance as associated factors of MSP were female sex (OR, 1.57; 95% CI, 1.07–2.29, *p* = 0.02) and LEDDs (OR, 3.35; 95% Cl, 1.63–6.59, *p* = 0.001). PD duration, MDS-UPDRS part III score in the OFF state and MDS-UPDRS part IV, PDSS (≥105 or <105), HAMD (≥8 or <8), and HAMA (≥7 or <7) scores were not significantly associated with MSP.

### Effects on QoL

Table 2 shows the results of the multiple linear regression analysis of variables associated with PDQ-39 scores in the patients with PD and MSP. The candidate variables were Hoehn and Yahr in the OFF state, MDS-UPDRS part III scores in the OFF state, MDS-UPDRS part IV scores, HAMD scores, HAMA scores, PD durations, MSP durations, and LEDDs. The result showed that MDS-UPDRS part III scores in the OFF state (β, 0.37; 95% CI, 0.26–0.48; *p* < 0.001), HAMD scores (β, 0.39; 95% Cl, 0.28–0.51; *p* < 0.001), and MSP durations (β, 0.17; 95% Cl, 0.06–0.28; *p* = 0.003) were significantly positively related to PDQ-39 scores. The MDS-UPDRS part IV, HAMA, PDSS, and NRS scores and ages were not related to PDQ-39 scores.

**Table 2:** Multiple linear regression analysis for variables associated with PDQ-39 scores in PD patients with MSP.

**Variables**	**β**	**95% CI**	***P-*value**
MDS-UPDRS part III (OFF state)	0.37	0.26–0.48	<0.001
HAMD	0.39	0.28–0.51	<0.001
Pain duration, years	0.17	0.06–0.28	0.003

*HAMD, Hamilton Depression Scale; MDS-UPDRS, Movement Disorder Society Unified PD Rating Scale; MSP, musculoskeletal pain; PD, Parkinson's disease; PDQ-39, PD Questionnaire*.

## Discussion

In the present study, we found that 45.58% of patients with PD experienced MSP. The distribution of MSP was variable and associated with various sensations. MSP was responsive to Levodopa therapy, and female sex and higher LEDDs were identified as risk factors for MSP. There was a significant association between longer MSP durations and reduced QoL.

We found that lower limbs and back pain were the most common sites. Similar results were reported in a previous study, demonstrating that MSP prevalence in specific body parts ranged from 20 to 60%. Among them, MSP in the back and lower limbs have the highest prevalence (15). Other studies showed that shoulder and back pain are the most common sites (2, 16). The reported prevalence of shoulder pain in PD ranged from 11 to 80% and that of back pain ranged from 59 to 74% (2). Some discrepancies between these studies, as mentioned above, are probably due to the different sample sizes of these studies.

Consistent with previous reports (2), Levodopa often alleviates MSP, and our findings reconfirmed this conclusion. Levodopa yielded decreases in pain scores in our MSP group, with 82.52% of the patients reporting pain relief after taking Levodopa, in good agreement with a previous study showing a high response rate (85%) to Levodopa therapy (17). Dopaminergic drugs are the mainstay of pharmacotherapy for patients with PD. However, the duration of pain relief achieved with dopamine therapy in some patients is short due to rapid dopamine metabolism; therefore, slow-release dopamine preparations are preferred and have been shown to be superior to oxycodone at providing sustained pain relief with minimal euphoria in patients with PD (18). Thus, it is evident that dopamine plays a significant role in PD with MSP. Though pathological changes in non-dopaminergic neurotransmitter systems seem to be implicated, dopaminergic denervation also provides a plausible substrate for altered central pain processing with increased pain sensitivity in PD (19).

As noted, female sex was a risk factor for MSP in our study; this observation is consistent with those of previous studies showing that women are more likely to have MSP across all age groups than men (7, 20). A sex-related pain mechanism may explain this. Chen et al. (21) reported that prolactin signaling contributes to pain and demonstrated that prolactin signaling promotes injury-free nociceptor sensitization in women. Another finding from Chen et al's research was that D2 dopamine receptor agonists could upregulate the prolactin receptor long isoform and prevent opioid hyperalgesia in women. Additionally, testosterone decreases pain sensitivity (22). Animal experiments showed that the microglia-neuronal signaling analgesic pathway involved in neuropathic pain is active only in male mice (23).

In addition to the female sex, LEDD was also a risk factor for MSP in our logistic regression analysis. A previous study reported LEDD as a related factor associated with higher pain scores but not a risk factor (24). In this previous study, the researchers concluded that LEDD was not a vital factor in all pain types with PD. However, they did not focus on MSP and omitted LEDDs in a further regression analysis, which may explain the inconsistencies with our results. Another previous study reported that MSP, PD duration, and higher LEDD were significant determinants of overall nocturnal sleep disorders (25). In this study, the researchers focus on sleep dysfunction. They did not analyze the risk factors of MSP in a further regression analysis, which may explain the inconsistencies with our results. Some patients with PD in late stages have marked loss of dopaminergic nigrostriatal neurons and are treated with lower LEDDs to avoid side effects. Still, based on our findings, we speculate that our MSP group patients might require a higher dose of Levodopa due to the more significant dopamine deficiencies. In other words, dopamine deficiencies might be a vital risk factor for MSP. Dopamine plays a crucial role in modulating nociception (26). Some studies have suggested that abnormal processing of nociceptive inputs can explain muscle pain in PD (27). Further, dopamine deficiencies are potential contributors to hyperalgesia (28). This hypothesis is supported by the fact that MSP was associated with LEDD but not the severity of motor symptoms, as assessed by the UPDRS part III score.

Furthermore, our analyses identified no relationship between MDS-UPDRS part III scores and MSP presence, and other studies also reported similar findings (6, 29). Some studies showed that MSP symptoms fluctuated in their response to dopaminergic treatment and were associated with the severity of motor symptoms, such as parkinsonian rigidity, akinesia, cramps, immobility, postural abnormalities, and dystonia (2, 4). Previous studies have revealed that motor symptoms and MSP were alleviated after taking Levodopa (2); thus, it is plausible to conclude that MSP results from the motor symptoms of PD. However, based on the previous study (6) and our findings, there is no apparent relationship between MSP and motor symptoms. Pain processing in the central nervous system may be more relevant to MSP in PD than to the effects of motor impairments (6, 27).

We also found no relationship between MDS-UPDRS part IV, HAMD, and HAMA scores, and MSP presence. This result conflicts with the findings of an earlier study (6), but the correlation between the severity of MSP and that of motor complications (*r* = 0.11), depression (*r* = 0.16), or anxiety (*r* = 0.2) reported in that study was weak. Furthermore, the previous study did not include additional analyses, such as logistic regression, which could be influenced by various confounding factors that affected the presence of MSP. This may explain the discrepancy between our results and those of the previous study. The lack of a relationship between motor symptoms and MSP is consistent with the observations reported for other pain conditions. For example, in complex regional pain syndrome, which can occur in various movement disorders, motor disorders are not associated with abnormal sensory plasticity (30). Therefore, we can infer that MSP may be independent of motor impairments but closely associated with dopamine deficiencies. To our best knowledge, our work is the first study to elucidate the interrelationship between MSP, motor impairments, and dopamine deficiencies.

The pain mechanisms in PD with MSP are complex. Previous studies have confirmed that MSP is associated with a decreased baseline availability of striatal dopamine D2/D3 receptors and a reduced level of presynaptic dopamine (31, 32). Descending dopaminergic pathways play an essential role in pain modulation (33). However, further research is required to determine the pain-generating mechanisms that account for MSP being more commonly experienced in some areas than in others. Some of the brain structures implicated in MSP in PD, such as the substantia nigra, amygdala, prefrontal cortex, thalamus, anterior cingulate cortex, insular cortex, and spinal cord, are essential for the transmission of nociceptive inputs and central pain processing and collectively form the pain matrix (2). Multiple neurotransmitters, such as opioids, glutamate, γ-aminobutyric acid, and dopamine, are involved in the modulation of pain by cortical structures in the pain matrix (34). Different signaling pathways and dopaminergic transmitters could mediate the transmission of pain signals in other brain structures (35, 36), and this may explain differences in the anatomical sites of MSP involvement. Furthermore, deep brain stimulation (DBS) has been suggested to relieve pain in patients with PD based on class III evidence (37, 38). However, another study reported controversial results that most patients who underwent DBS developed MSP (39). Therefore, the effect of DBS on MSP in patients with PD is unclear, and further studies are needed to address this issue.

Here, for the first time, we present, from these lines of evidence, that dopamine deficiencies, more than the motor symptoms, might underlie the development of MSP in PD. Further research is needed to test this hypothesis. Although the dopaminergic pain pathways are the dominant mechanisms of MSP based on the present and previous studies (40, 41), we also found that some patients were non-responsive or only partially responsive to dopamine therapy for their MSP. Jarcho et al. (40) indicated that the genetic influences on dopamine system function might affect pain; for instance, catechol-O-methyltransferase activity differentially affects the dopamine transmission in the medial prefrontal cortex and striatum. Exploring these pain mechanisms in PD with MSP would be a valuable line of future research.

Both the motor and non-motor symptoms of PD play critical roles in patients' perceptions of their QoL (42). In our analyses, the duration of MSP was significantly positively associated with low QoL in patients with MSP, but this association was weaker than that with other relevant variables, such as motor symptoms and depression. This conflicts with the findings of a previous study suggesting that pain of any subtype has a more significant effect on QoL in patients with PD than motor impairments (6). A possible reason for this discrepancy is that we focused on a single type of pain rather than all kinds of pain. However, our results highlight that the duration of MSP, in particular, has a substantial effect on the QoL of patients with PD. Indeed, MSP is highly prevalent and strongly associated with QoL in PD, making it an important target for treatment. To our knowledge, this is the first report to elucidate the variables related to QoL in PD patients with MSP. Further studies are needed to confirm our findings.

Our study has several limitations. First, although we ensured complete data documentation, it was not a randomized controlled study. Only two centers and patients with pain relieved by mobility or improved with Levodopa were included, which might result in selection bias. The actual prevalence of MSP might be higher than those reported in this study. Moreover, the related risk factors of MSP might be more than those reported in this study. Some secondary factors might be ignored, for instance, some of the nonmotor factors. Further studies with prospective, multi-center investigations with larger samples and a matched control population are required. Secondly, we did not collect data on autonomic symptoms, which are known to have a weak correlation with MSP (*r* = 0.17) (6). Thirdly, NRS may not be the most adequate tool because the assessment is not objective. Further studies should include pain assessment, such as a Sinicized King's PD pain scale or laser evoked potentials, more reliable tools to assess the functional state altered in patients with PD.

In conclusion, our study shows that MSP commonly occurs in patients with PD, affecting multiple body parts with various sensations. The most common MSP sites are the lower limbs and back. Levodopa therapy can alleviate MSP with an 82.52% response rate. Women and higher LEDDs are more likely associated with a higher risk of MSP than PD-related motor symptoms, which has substantially changed our understanding of PD with MSP. The observation that pain duration is an important determinant of QoL reinforces the need for future strategies aimed at improving MSP.

## Data Availability Statement

The raw data supporting the conclusions of this article will be made available by the authors, without undue reservation.

## Ethics Statement

Written informed consent was obtained from the individual(s) for the publication of any potentially identifiable images or data included in this article.

## Author Contributions

JL designed the study, interpreted the results, and wrote the manuscript. B-FZ designed the study, collected the data, and analyzed the data. S-SM, S-ZJ, and H-ZC collected the data. S-YL and CH advised the methodology and interpreted the results. Z-QG and HZ revised the manuscript. PC supervised the study, revised the manuscript, and is the study guarantor. All authors contributed to the article and approved the submitted version.

## Funding

This work was supported by the National Key R&D Program of China (Grant Nos. 2018YFC1312001, 2017YFC0840100, and 2017YFC0840105), Anhui University Science Research Project (Grant No. KJ2021A058), and the Outstanding Young Talents Project of Universities in Anhui Province Study Abroad (Grant No. gxgwfx2021028).

## Conflict of Interest

The authors declare that the research was conducted in the absence of any commercial or financial relationships that could be construed as a potential conflict of interest.

## Publisher's Note

All claims expressed in this article are solely those of the authors and do not necessarily represent those of their affiliated organizations, or those of the publisher, the editors and the reviewers. Any product that may be evaluated in this article, or claim that may be made by its manufacturer, is not guaranteed or endorsed by the publisher.
